# Prevalence of Food Insecurity among Women in Northern Jordan

**DOI:** 10.3329/jhpn.v30i1.11276

**Published:** 2012-03

**Authors:** Hiba A. Bawadi, Reema F. Tayyem, Amal N. Dwairy, Nemeh Al-Akour

**Affiliations:** ^1^Department of Nutrition and Food Technology, Jordan University of Science and Technology, PO Box 3030, Irbid 22110, Jordan; ^2^Department of Clinical Nutrition and Dietetics, Hashemite University, PO Box 150459, Zarqa 13115, Jordan; ^3^School of Nursing, Jordan University of Science and Technology, PO Box 3030, Irbid 22110, Jordan

**Keywords:** Body mass index, Cross-sectional studies, Food insecurity, Food security, Hunger, Women, Jordan

## Abstract

Food insecurity—not having sufficient quantities of good-quality foods—is inversely related to physical and mental health and directly related to poor dietary intake. The objectives of this research were to (a) measure the prevalence of food insecurity among women in northern Jordan, (b) study the socioeconomic factors associated with an increased risk of food insecurity, and (c) investigate the relationship between household food insecurity and women's reported body-weight. This cross-sectional study was conducted using an interview-based questionnaire. In total, 500 women were interviewed in the waiting rooms of the outpatient clinics of two major public hospitals in northern Jordan. Food insecurity was assessed using the short form of the U.S. food security survey module. The prevalence of food insecurity was 32.4%. Income below the poverty-line, illiteracy, unemployment, rented housing, and woman heading the household were among the socioeconomic factors that increased the probability of food insecurity. No evidence was found to support the relationship between obesity and food insecurity. Except grains, food-insecure women with hunger had lower intake of all food-groups. This study demonstrated that the problem of food insecurity is present in Jordan. Food-insecure women with hunger are at a risk of malnutrition. Interventions that target reduction of the factors associated with food insecurity are necessary.

## INTRODUCTION

Food security insures that all individuals in a household, at all times, have continuous access to enough good-quality food to support a healthy and productive life (1,2). Food security exists when all people, at all times, have physical and economic access to sufficient food to meet their dietary needs for a productive and healthy life. Food security has three dimensions: availability of sufficient quantities of food of appropriate quality, supplied through domestic production or imports; access by households and individuals to adequate resources to acquire appropriate foods for a nutritious diet; and use of food through adequate diet, water, sanitation, and healthcare (3). Hence, food insecurity is defined as lack of sustainable access to enough safe, nutritious, and socially-acceptable food. Food insecurity is a major factor contributing to hunger and malnutrition. Hunger and malnutrition have remarkable negative impacts on human health and productivity (4-6).

Like other developing countries, Jordan may have a significant proportion of its population classified as food-insecure. Jordan is a small country, with inadequate supplies of water and other natural resources. Debt, poverty, and unemployment are fundamental problems. Fourteen percent of the population of Jordan falls below the poverty-line of 392 Jordanian Dinar (US$ 553) per person per year. According to the World Food Programme (WFP), Jordan has several risk factors of food insecurity (7). The risk factors include: (a) lack of job opportunities; (b) degradation of agricultural land―the contribution from agriculture as a percentage of gross domestic product has decreased from 8% in 1990 to 4% in 1998; (c) self-insufficiency in food products―in the best of seasons, Jordan produces 8-10% of its cereal requirements; and (d) water scarcity in Jordan ranking among the five most water-deficit countries of the world (7). All these economic factors may lead to food insecurity in Jordan. Like every other country across the world, women are often the first to be caught in the problem of food insecurity in Jordan; no data are available on the state of food insecurity among women. Understanding the epidemiology of food insecurity among women in Jordan establishes basis for evaluating governmental and non-governmental efforts to reduce food insecurity. This study aimed at providing some data on the epidemiology of food insecurity among Jordanian women.

## MATERIALS AND METHODS

### Study subjects and data-collection

A cross-sectional survey was conducted among 500 women in Irbid, northern Jordan. Irbid was selected as the area of study because it has the second largest population in Jordan after Amman and has the highest density of population in the country. Irbid has high intra-population diversity. It has four universities. Irbid has an urban population of 707,420 and a rural population of 220,872. Moreover, it has two camps for Palestinian refugees. Based on these, Irbid is considered to have a representative Jordanian population from different socioeconomic classes (8). Women aged 18-70 years were selected from those who accompanied patients during their visit to the outpatient clinics of two major public hospitals in northern Jordan. Each day, for the whole study period (1 year), all women in the waiting rooms of the outpatient clinics of the two hospitals were asked to participate in the study. Women who agreed to participate in the study were asked to sign a consent form. A trained interviewer explained the research objectives and methods to each participant. Participants suffering from chronic diseases were not eligible to participate in the study to avoid any possible interaction between illness and food-security status. An interviewer-administered questionnaire was used for collecting data.

### Independent variables

**Intake of food groups:** A short food-frequency questionnaire was used for estimating the intakes of items from the following food groups; cereals, fruits, vegetables, meat, milk, and milk products. Most commonly-consumed items within each food group were included in the questionnaire. Information about serving-sizes for each item was provided and explained to the participants.

**Body mass index:** Body-weight and height were self-reported, and body mass index (BMI) was calculated as weight (kg) divided by the squared height (m^2^). The BMI cut-off points suggested by the World Health Organization were used.

**Age:** Women were asked to put themselves in one of the following age-categories: <35, 35-44, 45-55, and ≥55 years.

**Education:** Participants were asked to choose the highest level of education they have received. Choices provided were: no schooling, elementary, secondary, high school, and college education.

**Income:** Women were asked about the total household income earned by all household members. Several prevalence studies in Jordan used self-reported income as a measure of household income (9,10). Income was then divided by the number of household members to obtain the monthly income per head. The monthly income per head was compared with the national poverty-line in Jordan, and two categories were generated: less than or equal to the poverty-line and greater than the poverty-line.

**Other socioeconomic variables:** Women were also asked about the number of full-time employees in the household, including herself; person heading the household; number of children aged less than 21 years; and housing status (owned or rented).

### Dependent variable

The status of food insecurity was assessed using the short form of the U.S. food security survey module. Validation studies conducted in developing countries offered evidence that the module is valid and can be used for assessing different levels of food insecurity status in diverse developing world settings, such as Brazil, Korea, and Bolivia (11-13). Food insecurity level was classified as food insecurity without hunger (FIWOH) and the more severe form—food insecurity with hunger (FIWH). Classification was made based on the number of affirmative responses to the questionnaire. Households affirming zero or one item of the questionnaire were classified as food-secure; households affirming 2-4 items were classified as food-insecure without hunger; and households affirming 5 or 6 items were classified as food-insecure with hunger (14).

### Statistical analysis

Descriptive statistics, including frequencies, percentages, and cross-tabulations, were obtained to measure the distribution of food insecurity according to the different social and demographic factors. Odds ratios (ORs) were obtained to identify factors responsible for increasing the odds of food insecurity. Multiple logistic regressions were used for obtaining ORs. The ORs presented in the Results section estimated the risk of having FIWOH and FIWH with regard to the study variables (age, income, education, employment, head of household, housing status, number of children, and BMI). Comparison between women with different levels of food insecurity was made with regard to their average daily consumption of each food group. Means were subjected to univariate analysis using the general linear model of the SPSS software for Windows (version 16) (SPSS Inc., Chicago, IL, USA). The mean comparisons were analyzed using the least significant difference (LSD). The p value of <0.05 was considered significant.

## RESULTS

In total, 500 women completed the interview. Their average age was 37±10.8 years. Over half (53%) of the women were from families with monthly income of equal or greater than the national poverty-line. The mean income of the participants was 52±36 JD per month per head. The number of children (aged less than 21 years) living in the household ranged from 0 to 10. All the participants were apparently healthy, with the mean BMI of 27.7±5.

Of the 500 women, 338 (67.6%) were from food secure households, and 162 (32.4%) were from food, insecure households. Of the food-insecure households, 43% were suffering from hunger. Table 1 shows the responses obtained from the short form of the food-insecurity assessment module. About 33% of the women could not afford to eat balanced meals (meals containing all food-groups in the right proportions), and about 26% ate less than what they felt is enough due to lack of money.

Table 2 shows the prevalence of food insecurity according to age, income, education, employment, head of household, and housing. The ORs of being food-insecure were estimated for each variable. Both the types of food insecurity (with or without hunger) were not associated with women's age. Income was associated with the increased risk of both the types of food insecurity. Participants with income of less than the poverty-line were four times more likely to be food-insecure. Participants who rented houses had two times higher odds of being severely food-insecure when compared with those who owned houses.

**Table 1.** Responses to individual items on the food-security questionnaire (n=500)

Affirmative response to individual item	No.	%
In the last 12 months		
I cut the size of my meals or skipped meals because there was not enough money for food	104	21
If yes, how often did this happen? (almost every month/some months)	102	98*
I ate less than I felt I should because there was not enough money for food	131	26
I was hungry but did not eat because there was not enough money for food	84	17
The food that I bought just did not last, and I did not have money to get more (often/sometimes)	124	25
I could not afford to eat balanced meals (often/sometimes)	166	33

*Proportion calculated out of 104;

Respondents to this item only were those who responded ‘yes’ to the previous item

**Table 2.** Distribution of food insecurity according to social and demographic factors*

Variable	FIWOH No. (%)	Odds ratio (95% CI)	FIWH No. (%)	Odds ratio (95% CI)
Age (years)				
<35	37/222 (17)	1.3 (0.5-3.7)	22/222 (10)	1.0 (0.3-3.1]
35-44	31/146 (21)	2.0 (0.7-5.8)	26/146 (18)	2.1 (0.7-6.7]
45-54	19/93 (20)	2.0 (0.6-6.0)	18/93 (19)	2.4 (0.7-7.7]
≥55	5/39 (13)	-	4/39 (10)	-
Income				
<poverty-line	63/233 (27)	4.2 (2.5-6.9)	56/233 (24)	7.7 (4.1-14.5]
≥poverty-line	29/265 (11)	-	14/265 (5)	-
Education				
Illiterate	26/158 (17)	2.9 (1.1-7.6)	40/158 (25)	9.1 (2.7-30.8)
Elementary/secondary school	48/197 (24)	3.8 (1.5-9.3)	17/197 (9)	2.7 (0.7-9.5)
High school	12/71 (17)	2.5 (0.9-7.3)	10/71 (14)	4.2 (1.1-16.4)
College	6/72 (8)	-	3/72 (4)	-
Employment†				
0	5/19 (26)	7.3 (1.1-46.2)	8/19 (42)	9.1 (0.8-97.7)
1	46/327 (20)	1.2 (0.2-5.7)	48/327 (15)	3.2 (0.4-25.8)
2	18/109 (17)	0.5 (0.1-2.8)	8/109 (7)	2.3 (0.2-19.6)
3	4/29 (14)	1.0 (0.1-6.7)	4/29 (14)	2.1 (0.2-21.1)
≥4	1/14 (7)	-	2/14 (14)	-
Head of household				
Man	66/393 (17)	-	49/393 (13)	-
Woman	26/105 (25)	1.8 (1.1-3.2]	21/105 (20)	2.0 (1.1-3.6)
Housing				
Owned	76/427 (18)	-	54/427 (13)	-
Rented	16/71 (23)	1.6 (0.8-3.0)	16/71 (23)	2.2 (1.1-4.3)
Number of children				
0	14/71 (20)	-	8/71 (11)	-
1	5/47 (11)	0.5 (0.1-1.6)	8/47 (17)	1.4 (0.4-4.2)
2	16/86 (19)	0.9 (0.4-2.2)	12/86 (14)	1.2 (0.4-3.3)
3	10/72 (14)	0.7 (0.3-1.6)	10/72 (14)	1.1 (0.4-3.2)
≥4	46/221 (21)	1.1 (0.5-2.2)	32/221 (14)	1.3 (0.5-3.1)

*Figures are frequency (percentage);

†Number of full-time employees in the household;

CI=Confidence interval;

FIWH=Food insecurity with hunger;

FIWOH=Food insecurity without hunger

**Table 3.** Distribution of food insecurity according to BMI

BMI	FIWOH	Odds ratio (95% CI)	FIWH	Odds ratio (95% CI)
Underweight	0/12	Zero cell count	1/12	0.5 (0.1-4.6)
Normal	22/153	-	18/153	-
Overweight	37/170	1.7 (0.9-3.0)	21/170	1.1 (0.6-2.3)
Obesity	29/155	1.3 (0.7-2.5)	28/155	1.7 (0.9-3.4)

The World Health Organization's cut-off points were used for classification: underweight (BMI <18.5 kg/m^2^), normal weight (BMI 18.6-24.9 kg/m^2^), overweight (BMI 25-29.9 kg/m^2^), and obese (BMI ≥30 kg/m^2^). BMI=Body mass index;

CI=Confidence interval;

FIWH=Food insecurity with hunger;

FIWOH=Food insecurity without hunger

Level of education was associated with the food insecurity status. Participants who had less than 12 years of education were at a higher risk of being food-insecure compared to those who had a college degree. For instance, the ORs of food insecurity for illiterates compared to college degree-holders were 2.9 [95% confidence interval (CI) 1.1-7.6] and 9.1 (95% CI 2.7-30.8) for FIWOH (0-1 affirmative) and FIWH (2-4 affirmatives) respectively.

Having a woman as the head of a household was associated with both the types of food insecurity; the ORs were 1.8 (95% CI 1.1-3.2) and 9.1 (95% CI 1.1-2.6) for FIWOH and FIWH respectively. There was little evidence of linking the number of children (members aged less than 21 years) in the household with either type of food insecurity (p=0.08); however, none of the ORs was significant.

Table 3 shows BMI classification according to the level of food insecurity. None of the ORs was significant; hence, there was no significant association between food insecurity and body-weight among the Jordanian women. However, there was a trend of increasing odds of food insecurity among overweight (OR=1.7, 95% CI 0.9-3.5) and obese (OR=1.7, 95% CI 0.9-3.4) women.

The mean values for women's daily consumption of different food-groups are presented in Table 4. The food-secure women had the higher mean intakes of fruits, milk, and meat than those with food insecurity. With respect to grains, no differences in the intakes were observed among the food-secure women, FIWOH, and FIWH (p=0.2). The food-secure women consumed higher quantities of fruits than women in the FIWOH and FIWH groups. The food-secure women consumed higher quantities of vegetables, milk and milk products, and meat compared to the food-insecure women with hunger.

**Table 4.** Comparison of women's servings per day from each of the food-groups with their household food-security status (mean±SD)

Food-group	Food-secure	FIWOH	FIWH
Servings*	n=328	n=91	n=70
Cereal	3.7±0.05^a^	3.8±0.1^a^	3.5±0.12^a^
Vegetables	1.3±0.03^a^	1.2±0.06^a,b^	1.1±0.07^b^
Fruits	1.2±0.7^a^	0.9±0.7^b^	0.5±0.4^c^
Milk and milk products	1.5±0.05^a^	1.3±0.09^a,b^	1.1±0.1^b^
Meat	3.2±0.09^a^	3.1±0.2^a^	2.3±0.2^b^
Sweets	1.6±0.5^a^	1.4±0.1^a^	1±0.1^b^

Values within the row with different superscripts are significantly different. The level of significance was p<0.05.

*Serving-sizes used are the foods guide pyramid serving-sizes;

FIWH=Food insecurity with hunger;

FIWOH=Food insecurity without hunger;

SD=Standard deviation

## DISCUSSION

Approximately one-third (32.4%) of the women in northern Jordan were food-insecure. This percentage is considerably higher than that found for women in developed countries; for instance, only 18% of women were food-insecure in the USA (15). The association found between income and food insecurity is consistent with that reported in other studies (16-19). Other socioeconomic variables that were associated with the increased risk of food insecurity included level of education, head of household, and ownership of house.

In the present study, women who did not hold a college degree were more likely to be suffering from hunger. A recent report of the European Training Foundation (ETF) documented that employment of Jordanian women increased with level of their education (20). Thus, there are increased rates of unemployment among women without a college degree. Of employed Jordanians, 55% of men have an educational level lower than secondary while over two-thirds of female workers have a higher educational qualification (37% have a bachelor's degree or higher and 31% a college or intermediate diploma) (20). In Jordan, professional jobs are more culturally acceptable, especially for women. In most cases, these kinds of jobs require higher level of education. According to the ETF report, occupational distribution by gender reveals that women are over-represented in certain occupations, particularly professional and technical occupations, and are under-represented in other occupations (labour-workers in craft, etc.) (20). In other words, Jordanian women are able to get culturally-acceptable jobs in most cases, only if they have a higher level of education. Otherwise, they tend to be unemployed, thereby having low income and are more prone to food insecurity. On the other hand, this scenario is not applicable to men, where employment status is not necessarily dependent on level of education. Culturally speaking, men have more choices of jobs that do not require higher level of education; thus, “having a man as the head of household” appears to be a common protective factor against food insecurity. Gulliford *et al.* reported the same trend (4).

The relationship between food insecurity and gain in weight has been studied but the magnitude and nature of the relationship are still not clear. The results of our study suggest a trend of a relationship between body-weight and the state of food insecurity. All the ORs obtained for overweight and obesity (considering normal weight range as a reference) were greater than 1 for both the types of food insecurity. However, none of the ORs was significant. Olson studied this relationship among 193 low-income women. The study found that women who suffered from mild food insecurity had higher BMI than food-secure women (21). The same association in women was reported later by Townsend *et al.* using secondary data from the 1994-1996 Continuing Survey of Food Intakes by Individuals (CSFII) (22).

On the other hand, results of a cross-sectional study by Sarlio-Lahteenkorva and Lahelma in Finland revealed that only thinness was associated with severe food insecurity (23). The association was not clear for overweight and obesity; however, obese individuals reported more fear of running out of money to buy foods due to economic problems during the past 12 months. Gulliford *et al.* studied the relationship between food insecurity and body-weight in 241 men and 290 women (4). The study did not find any association between food insecurity and obesity (4). Another study by Jones and Frongillo did not also find any evidence that supports a relationship between food insecurity and overweight (24). These conflicting findings highlight the need for further studies, with enough power, to elaborate the nature of the relationship between food insecurity and gain in weight.

In our study, the intakes of fruits, milk, and meat were higher among the food-secure women than among the food-insecure women with hunger (p<0.05). In another study, food-insecure individuals were less likely to report “eating fruits and vegetables at least 5-6 days a week” compared to food-secure individuals (4). The consumption of fruits was positively related to income. In a study on preschool children, recipients of food stamps with low income reported significantly lower consumption of fruits (25). No difference was found in terms of consumption of grains. Grains (wheat and rice) are considered staple foods in Jordan. Prices are low as the Government co-pays the cost, along with the consumer (26).

In summary, food insecurity is prevalent among women in northern Jordan. The finding that about one-third of the North Jordanian women are food-insecure is alarming. The factors that had impact on food insecurity in northern Jordan included: income, education, employment, and women heading the household. No evidence was found in the present study to support the association between food insecurity and the increased risk of overweight and obesity. The consumption of fruits decreased as the severity of food insecurity increased.

### Limitations

This study is primarily limited due to self-reported data with regard to height and weight. Other limitations include cultural shame associated with declaring low economic status and inability to afford adequate nutrition.

### Conclusions

Food insecurity and hunger are common among North Jordanian women. Further studies are urgently needed to measure food insecurity nationwide. It is time for health-promotion professionals and policy-makers in Jordan to plan interventional programmes to reduce the magnitude and consequences of the problem. Several socioeconomic factors are linked to a higher risk of food insecurity, including income below the poverty-line, illiteracy, unemployment, rented housing, and woman heading the household. More efforts should be directed towards increasing the level of literacy among Jordanian women. Women's literacy is the key to empowering women and to improving families’ well-being. The intake of fruits, milk, and meat was low by those who suffered from food insecurity with hunger compared to the food-secure women. More studies with a larger sample-size and women from all over the kingdom are needed to determine the prevalence and risk factors of food insecurity among Jordanian women.

## ACKNOWLEDGEMENTS

The authors thank the Deanship of Research at the Jordan University of Science and Technology, Irbid, Jordan, for providing financial support for this project. The collaboration of the volunteers is also greatly appreciated.
